# Predictive factors for response to salvage stereotactic body radiotherapy in oligorecurrent prostate cancer limited to lymph nodes: a single institution experience

**DOI:** 10.1186/s12894-019-0515-z

**Published:** 2019-09-09

**Authors:** Christoph Oehler, Michel Zimmermann, Lukas Adam, Juergen Curschmann, Marcin Sumila, Räto T. Strebel, Richard Cathomas, Qiyu Li, Uwe Schneider, Daniel R. Zwahlen

**Affiliations:** 1 Department of Radiation Oncology, Kantonsspital Graubünden, Loëstrasse 170, 7000, Chur, Switzerland; 2 Center for Radiation Oncology, Hirslanden Klinik, Zürich, Switzerland; 3 Department of Urology, Kantonsspital Graubünden, Chur, Switzerland; 4 Division of Medical Oncology, Kantonsspital Graubünden, Chur, Switzerland; 5 Statistics Unit, SAKK Coordinating Center, Bern, Switzerland

**Keywords:** Body radiotherapy, Stereotactic, Prostate cancer, Lymph node metastases, Salvage therapy, Recurrence

## Abstract

**Background:**

In patients presenting with limited nodal recurrence following radical prostatectomy (RP), stereotactic body radiotherapy (SBRT) results might improve with a better case selection.

**Methods:**

Single-institution retrospective analysis of patients presenting with 1–3 lymph node (LN) recurrences (N1 or M1a) on 18F-Choline PET/CT. Prior therapy included radical prostatectomy (RP) ± salvage radiotherapy (RT), in absence of any systemic therapy.

Outcome parameters were biochemical response (BR), time to biochemical recurrence (TBR) and time interval between SBRT and androgen deprivation therapy start (TADT). Time to event endpoints was analysed using Kaplan-Meier method. Potential prognostic factors were examined using univariate proportional hazards regression for TADT and logistic regression for BR. The optimal cut-off point for LN size was calculated using the Contal and O’Quigley method.

**Results:**

25 patients fulfilling study criteria were treated with SBRT from January 2010 to January 2015 and retrospectively analysed. Median follow up was 18 months and median LN diameter 10.5 mm. SBRT was delivered to a median dose of 36 Gy in three fractions (range: 30–45 Gy). BR was reached in 52% of cases. Median TBR was 11.9 months and significantly longer in patients with larger LN (Hazard ratio [HR] = 0.87, *P* = 0.03). Using 14 mm as cut off for LN, median TBR was 10.8 months for patients with small LN (18 patients), and 21.2 months for patients with large LN (6 patients) (P unadjusted = 0.009; *P* adjusted = 0.099). ADT was started in 32% of patients after a median follow-up of 18 months.

**Conclusions:**

For PCa patients with 1–3 LN recurrence after RP (± salvage RT), SBRT might result in a better biochemical control when delivered to larger sized (≥ 14 mm) LN metastases. This study is hypothesis generating and results should be tested in a larger prospective trial.

**Electronic supplementary material:**

The online version of this article (10.1186/s12894-019-0515-z) contains supplementary material, which is available to authorized users.

## Background

Prostate cancer (PCa) patients diagnosed with biochemical recurrence and limited metastatic disease are conventionally treated with androgen deprivation therapy (ADT). Recently, stereotactic body radiation therapy (SBRT) has emerged as a promising approach for oligometastatic PCa recurrence. This strategy might have the potential to defer disease progression, thereby lengthening the time till initiation of hormonal therapy, and thus improving quality of life.

Results from a recent prospective phase II study (STOMP trial) suggest that metastasis-directed therapy (surgery or SBRT) of up to three extracranial metastases (any N1 or M1) benefited patients by postponing the start of ADT when compared to surveillance (median ADT-free survival of 21 months and 13 months, respectively) [1]. In a pilot phase I/II study (POPSTAR trial), SBRT delivered to 1–3 metastatic lesions (bone or LN) resulted in a 2-year ADT-free survival rate of 48% among 22 patients who did not have ADT at the time of SBRT [2]. Results from the promising ORIOLE phase II trial (52 patients randomized 2: 1 to SBRT or observation, combined with exhaustive translational research) are not expected before 2022 [3].

Retrospective series published are quite heterogeneous regarding the location of treated metastases (including various amounts of bone, nodal or visceral locations), the irradiated volume for patients with nodal disease (involved-nodes only, or associated to a prophylactic irradiation of the pelvic lymph drainage), and the initial radical PCa treatment (mixing radical prostatectomy (RP) ± radiotherapy (RT) with “RT only” or brachytherapy cases) [4].

The aim of this single-institution study was to investigate a homogenous population of exclusively ADT-naïve prostate cancer patients following RP ± salvage RT, presenting with one to three metachronous LN metastases on 18F-Choline PET/CT (CholPET/CT) and treated by SBRT. Endpoints of interest were biochemical response (BR), time to biochemical recurrence (TBR), and time interval between SBRT and ADT start (TADT).

## Methods

### Design and participants

This retrospective study obtained ethical approval. Patients provided written consent for SBRT treatment and for the use of their anonymized data for research purposes (ClinicalTrial.gov Identifier NCT03604211).

Inclusion criteria were as follows: histologically proven diagnosis of PCa; prior treatment with RP and pelvic lymphadenectomy (± salvage RT limited to the prostatic bed only), followed by a PSA relapse, defined by two consecutive rising PSA values > 0.2 μg/l [5]; maximum one to three N1 or M1a lesions (CholPET/CT positive) with a controlled primary tumor (CholPET/CT negative); WHO performance status of 0–1; no prior chemotherapy or ADT for PCa (castration-naïve patients).

Exclusion criteria included a primary treatment for PCa by RT or brachytherapy to achieve a more homogeneous patient cohort. PCa patients after RP experience actually a disturbed lymph drainage, with a high occurrence of aberrant LN spread [6], which has not been described after primary RT or brachytherapy, as far as we are aware of. Furthermore, primary RT for high-risk PCa is often combined with pelvic LN irradiation, which might lead to delivering non-ablative SBRT for nodal recurrences in the obturator and iliac basins, to respect the dose constraints for organs at risk. Similarly, patients having received salvage RT of the prostatic bed combined with pelvic nodal irradiation were excluded.

Further exclusion criteria were the occurrence of > 3 LN lesions, or bone (M1b) or visceral (M1c) metastases, or any symptomatic nodal lesion.

LN were considered as positive on CholPET/CT if either their uptake was markedly increased (higher than normal liver uptake) or if their uptake was moderately increased, and their size and shape did not indicate a clear benign LN. For our study, the reference diameter was based on the long-axis of positive LN; small-axis diameters were often not mentioned in nuclear medicine records. Measurements were performed on intravenous contrast enhanced CT images collected during each CholPET/CT examination.

PSA doubling-time (PSADT) before SBRT was calculated using the natural log of 2 divided by the slope of the linear regression line of the natural log of PSA against time (months) [7]; results were stratified according to three distinct PSADT categories: < 3, 3–6 and > 6 months.

During follow-up after SBRT, patients were reviewed clinically at approximately 3 to 6 month intervals by their urologist; PSA testing and imaging frequency was left up to the discretion of the physician in charge.

For patients experiencing radiological progression after SBRT, recurrence was considered as in-field, marginal or out-of-field, if it was located inside, at the border or outside the initial planning target volume (PTV).

ADT was started after SBRT for patients with radiological progression of baseline-detected soft-tissue lesions using the Response Evaluation Criteria in Solid Tumours [8], or detection of new bone or soft tissue metastases, as long as they were not amenable to further RT. PSA progression only was generally not an indication to start ADT.

### SBRT treatment

All patients were treated with SBRT delivered by CyberKnife®. Patients were immobilized using a customized external vacuum-type cast (CIVCO Medical Solutions, Kalona, IO, USA) prior to their CT planning scan (without IV contrast), acquiring images of 1.5 mm slice thickness.

Dose and fractionation were dependent on whether the patient had received previous external beam RT and normal tissue tolerances within the area to be treated. The prescribed doses ranged between 30.0 Gy and 45.0 Gy in three fractions, and were defined by the treating radiation oncologist. The prescribed dose was unrelated to LN size and increased progressively during the study period with growing experience. Treatments were designed using Multiplan software (Accuray, version 2.0.5). The planning target volume (PTV) was generated with 2–4 mm margin around the gross tumor volume (GTV).

SBRT was delivered every other day, using a non-isocentric treatment technique. Patient movement was monitored using the CyberKnife® intrafractional image guidance solution using implanted fiducial markers and relevant corrections applied.

### Endpoints

Endpoints of interest were biochemical response (BR), time to biochemical recurrence (TBR), and time interval from SBRT to ADT start (TADT).

PSA reduction in percent was calculated as follows: 100 x [PSA (ug/l) at SBRT- PSA (ug/l) at nadir post-SBRT/PSA (ug/l) at SBRT].

BR was defined as a reduction in PSA level by more than 50% of the baseline value, biochemical progression as a PSA increase ≥25% and ≥ 2μg/l if baseline PSA ≥ 2 μg/l, or a PSA increase ≥2μg/l if baseline PSA < 2 μg/l [9].

BR was also defined according to Jereczek criteria, with PSA cut-offs of + 10% (progression), − 10% (minor response) and − 50% (major response) [10].

TBR was defined as the time interval between SBRT until second consecutive PSA rise above reference PSA value. For patients responding to SBRT, the reference PSA was defined as the PSA nadir after SBRT. For patients not responding to SBRT, the reference PSA was the last PSA before SBRT (baseline PSA value).

TADT was defined as the time between SBRT and the start of palliative ADT or death of any cause.

Toxicity data were not evaluated; follow-up after SBRT was mostly performed by urologists, and toxicities not systematically assessed and reported.

### Statistical analysis

Descriptive statistics were used to summarize patient characteristics; results are expressed as median ± interquartile range (IQR). BR was calculated based on the maximum percentage change in PSA from baseline; it is reported for each patient using a waterfall plot. Time to event endpoints were analysed using Kaplan-Meier method. Time intervals were calculated from SBRT start to the event of interest. Censoring was at the date of last clinical follow-up in those not known to be deceased. Potential prognostic factors including age, last PSA and PSA doubling-time before SBRT, radiation dose, largest size and number of LN treated, as well as pathology results dating back to the initial prostatectomy, salvage RT after RP or not, were examined using univariate proportional hazards regression for TADT and logistic regression for BR. The optimal cut-off point for LN size was calculated using the method proposed by Contal and O’Quigley [11]; it is based on the log rank test statistic, and allows to identify a cutpoint when the outcome of interest is measured as time to event. The differences in survival between the groups were compared by the log-rank test. Statistical analyses were performed with R version 3.2.3.

## Results

### Patients

Thirty-eight patients treated with SBRT for oligometastatic recurrence following primary treatment of localized PCa were identified over a five-year period (January 2010 to January 2015) and entered into study. Thirteen patients did not meet all our inclusion criteria and had to be excluded. Three cases did not get RP as primary treatment [brachytherapy (*n* = 1) and external beam RT (*n* = 2)]. Six cases received ADT before SBRT, and another four were lost to follow-up (international patients residing outside the country). Twenty-five patients were included in the analysis.

Initial findings at RP are shown in Table 1. According to d’Amico classification [12], seven (28%) patients had intermediate risk disease and 18 (72%) high risk disease. The median time interval between RP and nodal SBRT was 35 months (range: 8–157). Baseline characteristics at SBRT are summarized in Table 2. Two thirds (*N* = 16, 64%) of patients had only one positive LN on CholPET/CT, with a total of 36 LNs treated for 25 patients, one third (*N* = 9, 36%) of patients had 2–3 positive LN on CholPET/CT. Location and size of involved LN are provided in Table 2 and (Additional file 1: Table S1). Median follow-up after SBRT was 18 months (IQR: 14–22). Three patients received a second SBRT treatment: one patient with infield relapse (obturator LN) and two cases of out-of-field relapse (obturator LN = > internal iliac LN; common iliac LN = > para-aortal LN; one each). Time interval between both SBRT was 8, 13 and 14 months, respectively.

**Table 1:** Patient and tumor characteristics (*n* = 25 patients)

Characteristic	Value	
Initial treatment		
RP	10	40%
RP + salvage RT	15	60%
Pre-operative PSA (μg/l)^a^		
median (IQR)	9.1	(5.4–20.4)
mean (range)	12.7	(1.8–40.5)
Gleason score		
6	1	4%
7	13	52%
8	7	28%
9	4	16%
T stage		
pT2	11	44%
pT3	14	56%
ECE		
no	13	52%
yes	12	48%
SVI		
no	19	76%
yes	6	24%
SM		
negative	13	52%
positive	12	48%

^a^ one data missing (*n* = 24)

**Table 2:** Patient and SBRT treatment characteristics (*n* = 25 patients)

Characteristics	Value	
Age at SBRT (years)		
median (IQR)	68	(62–72)
mean (range)	68	(52–81)
PSA at SBRT (ug/l)		
median (IQR)	3.53	(1.48–6.86)
mean (range)	3.49	(1.02–14.54)
PSADT		
< 3 months	5	20%
3–6 months	12	48%
> 6 months	8	32%
Number of lymph nodes at SBRT		
1	16	64%
2	7	28%
3	2	8%
LN localisation (*n* = 36)		
N1	33	92%
M1a	3	8%
Largest LN diameter (mm)^a^		
median (IQR)	10.5	(8.5–13.5)
mean (range)	12.8	(5–33)
SBRT data (Gy)		
total median dose (range)	36	(30–45)
median dose per fraction (range)	12	(10–15)
SBRT regimen		
10 Gy per 3 fractions	2	8%
11 Gy per 3 fractions	1	4%
12 Gy per 3 fractions	11	44%
13 Gy per 3 fractions	7	28%
15 Gy per 3 fractions	4	16%

^a^ one data missing (*n* = 35)

### Outcomes

#### Biochemical response (BR)

**Fig. 1:**
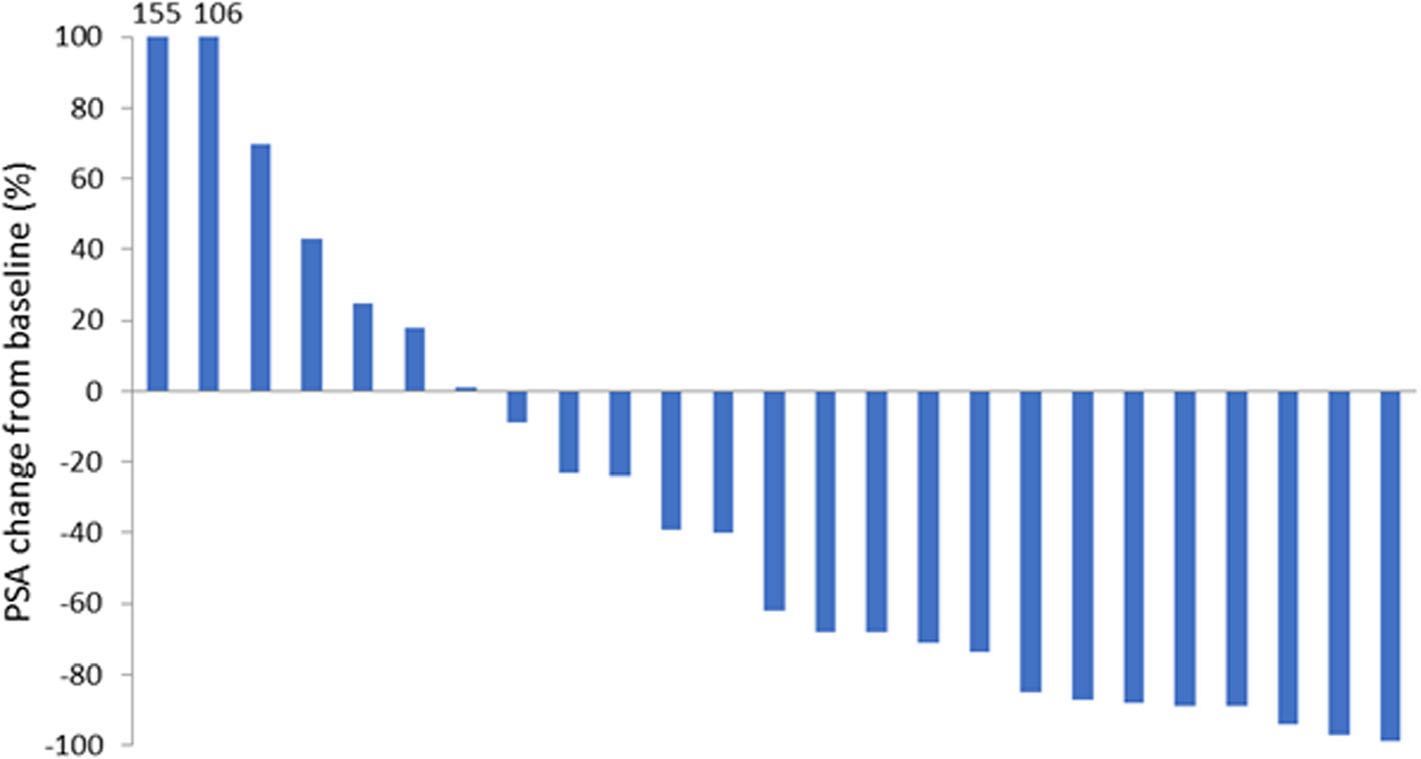
Best biochemical response after SBRT

Results are provided as a waterfall plot on a per-patient basis (Fig. 1). BR was observed in 13 patients (52%), while five patients (20%) experienced biochemical progression.

According to the response criteria from Jereczek et al., 17 patients (68%) experienced a PSA drop by at least 10%, 13 patients (52%) by more than 50%. On the other hand, PSA progressed by more than 10% in six patients (24%).

#### Time to biochemical recurrence (TBR)

**Fig. 2:**
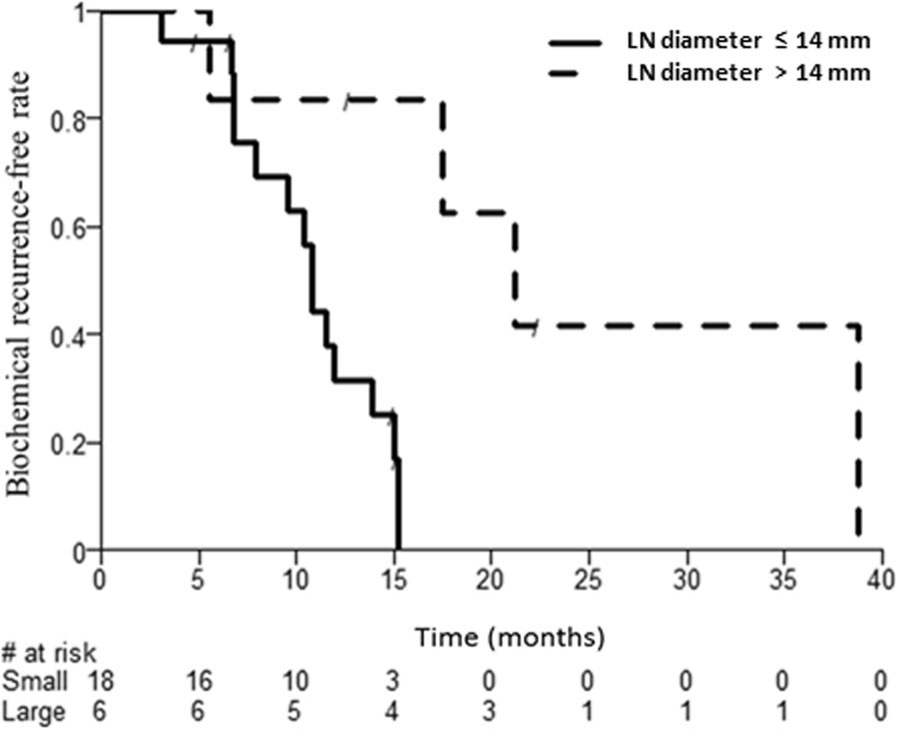
Time to biochemical recurrence after SBRT according to LN size (≤ 14 mm vs. > 14 mm)

**Fig. 3:**
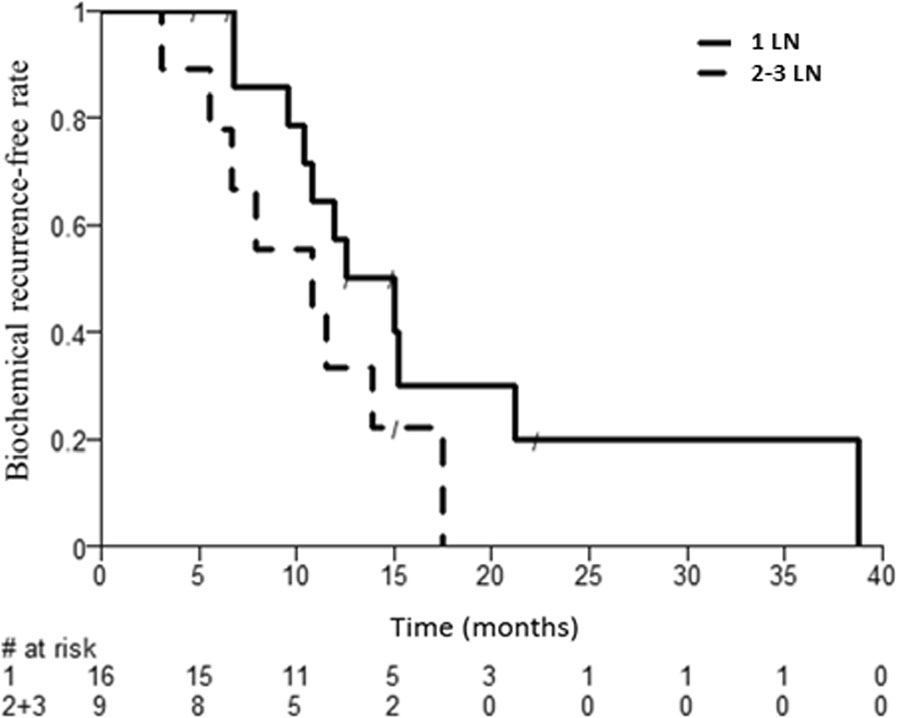
Time to biochemical recurrence after SBRT according to LN number (1 vs. 2–3)

Results from the univariate Cox regression analysis of the potentially prognostic factors on time between SBRT and second PSA rise are presented in (Additional file 1: Table S2). The risk of having a second PSA rise after SBRT decreased with the size of the largest LN, with a HR of 0.87 ([95% CI: 0.77–0.99]; *P* = 0.030). In our study, a LN size of 14 mm corresponded to the optimal cut-off, based on the Contal and O’Quigley method [13]. Median TBR was 10.8 months for patients with small LN, and 21.2 months for patients with large LN (P unadjusted = 0.009; *P* adjusted = 0.099). Kaplan-Meier curves of TBR stratified by LN size and number of LN are provided as Figs. 2 and 3.

#### Time to ADT

After a median follow-up of 18 months, 68% of patients remained free of any hormone therapy; ADT was started in 8/25 cases (32%) during the follow up period.

In a further analysis, we identified T3 stage (HR 9.34 [1.12–77.73] *P* = 0.039) and extra-prostatic extension at surgery (HR 10.29 [1.25–84.78] *P* = 0.030) as risk factors for a shorter TADT (Additional file 2: Figures S1 and S2). The influences of the Gleason score and PSA doubling-time on TADT were statistically not detectable in this small retrospective cohort (*P* = 0.23 and *P* = 0.47 respectively).

## Discussion

To the best of our knowledge, this is one of the largest retrospective series on LN recurrence treated with ablative SBRT exclusively in ADT-naïve prostate cancer patients. A biochemical response was observed in 52% of patients. After a median follow-up of 18 months, 68% patients were free of ADT, and only one of 36 treated LN (2.8%) required a second SBRT for in-field progression.

Jereczek-Fossa et al. recently reported on a cohort of 94 patients treated with salvage SBRT with a similar follow-up of 18.5 months [13]; initial treatment of prostate cancer was RP (42%), RP + RT (36%), RT alone (20%) and brachytherapy (2%), with or without ADT. In our series, PSA response, stabilization and progression after SBRT were observed in 68, 8 and 24% of cases, respectively. Our results replicate the 67, 16 and 18% reported by the colleagues from Milan, using similar biochemical response criteria. During the study period, ADT was started in 32% of our patients and in 36% in Milan [13]. Interestingly, for those receiving ADT after SBRT during the study period, treatment could be deferred by at least 1 year in 63% of our cases but only in 38% by the Milan group. Potential explanations for a later ADT start in our institution might include differences in ADT prescription practices or in tumour biology (8% M1a LN against 39.5% in Milan), but might just reflect higher median dose prescriptions (3 × 12 Gy in our center versus 3 × 8 Gy in Milan), corresponding to a twice higher biological equivalent dose (BED) with an α/β = 2 [14, 15] for prostate cancer (BED_2_ of 252 Gy versus 120 Gy). The latter BED might not be ablative for all LN metastases, and would provide a reasonable explanation for the 10% rate of in-field progression observed by Jereczek-Fossa et al.

In prostate cancer, conventional imaging has very poor accuracy for identifying LN metastases. In a meta-analysis including 24 studies, sensitivity was 42% for CT and 39% for MRI, specificity 82% for both [16]. Integration of CholPET/CT into routine evaluation of PCa patients improved both staging accuracy and RT planning [17, 18]. The detection rate of CholPET/CT is higher in cases of high PSA velocity (> 5 μg/l/yr) and short PSA doubling time (< 2 mo or 3 mo). Detection rate falls to < 30% for PSA levels < 1 μg/l, but rises > 50% for PSA levels > 2 μg/l [19]. Kitajima et al. reported a 75% detection rate for a PSA level between 4 and 10 μg/l [20].

Prostate-specific membrane antigen (PSMA) PET was not available at the time of our study. PSMA PET outperforms CholPET/CT for the localization of disease sites at biochemical recurrence, particularly in small lymph nodes and at lower PSA levels (< 1 μg/l). [21]. Many PSMA PET positive lesions present actually without an anatomic correlation on CT or MRI, indicating that focal treatment can be initiated at an early stage of metastatic disease [21].

It remains unclear if PSMA PET instead of CholPET/CT guided salvage treatment would have had a major impact on our results. PSA levels before SBRT were quite high in our series (median PSA: 3.53 μg/l); it is therefore likely that most positive LNs were correctly identified on CholPET/CT.

This elevated PSA before SBRT rises also the issue of delayed referral to CholPET/CT, with the advantage to better select patients with true oligo-recurrent disease for SBRT, but at the cost of exposing other patients to a higher risk of delayed treatment for more advanced progressive disease. This selection bias appears to discriminate elderly patients, as there are only two octogenerians in our series (8%). We hypothesize that 80+ year patients are mostly followed up with PSA and CT scans, and receive ADT instead of a metastasis-directed therapy at the time of nodal progression.

We found that salvage SBRT for isolated LN recurrent PCa provides a twice longer biochemical control (21.8 months vs. 10.8 months) when delivered to patients presenting with larger LN metastases (≥ 14 mm). On the other hand, the common clinico-pathological risk factors (T3, Gleason 8–10, PSA doubling time < 3 months) didn’t lead to a shorter TBR (Additional file 1: Table S2). At first glance, such results appear counterintuitive, as one would expect larger LN to reflect a higher tumor burden and a more advanced stage of disease, leading to a shorter TBR. These patients with 1–3 LN macrometastases, despite high-risk features at the time of RP, exhibit a phenotype of slow lymphatic spreading, with only a few LN metastases growing up over time without developing multiple new lesions (true oligometastatic disease). Given the retrospective nature of the study design, we hypothesize that our data result from a selection bias, selecting tumors that didn’t develop yet multiple mechanisms of immune evasion. Identifying the underlying gene expression pattern in such patients would allow in the future for a better selection of candidates to metastasis-directed SBRT, and for an earlier start of treatment (PSA < 1.0 μg/l and LN size < 1 cm). MicroRNA profiling shows substantial potential to be used as prognostic biomarker for individuals with oligometastatic prostate cancer [22–25].

The main strength of this study relies on quite restrictive inclusion criteria, leading to a series of 25 consecutive patients with a limited nodal recurrence (1–3 LN) after RP, in the absence of any prior or concomitant ADT. Regarding treatment, SBRT was always delivered at ablative doses (median dose: 36 Gy/ 3 fractions [range: 30–45 Gy]).

Our series has several limitations. The number of included patients was low, limiting the validity of subgroup analysis; observed results could have occurred merely by chance. Imaging and PSA levels during follow-up after SBRT were not obtained at predefined time points. We didn’t stage our patients with PSMA-PET, which has been shown in multiple studies to be superior to CholPET/CT imaging in the setting of biochemical relapse [26]. Further drawbacks include the lack of confirmatory biopsy for PET-positive LN, the absence of radiological response assessment after SBRT, and the lack of a control group. Time to ADT might also have been subscriber biased due to the lack of predefined prescription criteria. Finally, our results might not apply to patients with nodal metastases occurring with concomitant local recurrence (prostate bed) and/or distant (bone) metastases, nor to patients with castrate-resistant prostate cancer presenting with oligo-progressive disease.

Last, for patients experiencing isolated metastatic LN recurrence after primary treatment, there are currently no consensus guidelines on which strategy to follow. Besides treating only positive LN with SBRT, which could be considered in one sense as “just targeting the tip of the iceberg”, more comprehensive strategies might be applied as well, like salvage radiation of the whole pelvic lymph node basin combined with a simultaneous integrated boost (SIB) to PET-positive LNs, using either CholPET/CT [27] or PSMA PET/CT guided RT [28]. Extended salvage lymph node dissection (sLND) might be a valid option for selected patients, but remains technically challenging: sLND alone is usually not associated with a durable biochemical response; 50–80% of patients will receive some kind of adjuvant treatment right after sLND (ADT or RT) regardless post-operative PSA levels [29]. In an interesting retrospective analysis of 93 patients, adjuvant RT after sLND was shown to improve significantly the 5 year- relapse-free survival from 15.4% in the surgery only group to 34.3% in the group with additional RT (*P* = 0.0122) [30]. Prospective randomized trials are warranted to confirm these encouraging results.

## Conclusions

The present work underlines the possible role of ablative SBRT in the management of patients affected by LN oligo-recurrent disease. In ADT-naïve prostate cancer patients with 1–3 LN metastases after definitive RP, SBRT can offer a good control of asymptomatic disease. After a median follow-up of 18 months, 68% of patients remained free from ADT. Our study is the first to date attempting to define an optimal LN size threshold prior to SBRT. Additional studies are required before the results should be used in usual clinical settings.

## Additional files

Additional file 1: **Table S1.** Lymph node localisation before SBRT (*n* = 36). **Table S2.** Univariable cox regression on time interval from SBRT to second PSA rise. (DOCX 16 kb)

Additional file 2: **Figure S1.** Time to ADT after SBRT according to initial T stage (pT2 vs. pT3). **Figure S2.** Time to ADT after SBRT according to initial presence/absence of ECE. (ZIP 106 kb)

## Data Availability

The datasets analysed during the current study are available from the corresponding author on reasonable request.

## Abbreviations

ADT: Androgen deprivation therapy
ECE: Extra-capsular extension
IQR: Interquartile range
LN: Lymph node
PSA: Prostate specific antigen
PSADT: PSA doubling time
RP: Radical prostatectomy
RT: Radiotherapy
SBRT: Stereotactic body radiation therapy
SM: Surgical margins
SVI: Seminal vesicles involvement
