# Supplementary material for: Predictive factors for response to salvage stereotactic body radiotherapy in oligorecurrent prostate cancer limited to lymph nodes: a single institution experience
Source: BMC Urol. 2019 Sep 9;19:84. doi: 10.1186/s12894-019-0515-z (PMC6734440; doi:10.1186/s12894-019-0515-z)
Supplement: Supplementary file 1 — Table S1. Lymph node localisation before SBRT (n = 36). Table S2. Univariable cox regression on time interval from SBRT to second PSA rise. (DOCX 16 kb) [file 12894_2019_515_MOESM1_ESM.docx]

**Table S1. Lymph node localisation before SBRT (n = 36).**

|  |  | |
| --- | --- | --- |
| Localisation | Number | |
|  |  |  |
| **N1** | **33** |  |
| obturator | 12 | 33% |
| internal iliac | 6 | 17% |
| external iliac | 10 | 28% |
| common iliac | 4 | 11% |
| para-rectal | 1 | 3% |
| presacral | 0 | 0% |
|  |  |  |
| **M1a** | **3** |  |
| para-aortal | 1 | 3% |
| Ilio-psoas | 2 | 5% |
|  |  |  |

**Table S2.** **Univariable cox regression on time interval from SBRT to second PSA rise.**

|  |  |  |
| --- | --- | --- |
|  | HR (95% CI) | P-value |
|  |  |  |
| Age | 1.02 (0.94-1.10) | 0.647 |
| Baseline PSA | 1.07 (0.92-1.24) | 0.392 |
| PSA doubling time | 0.97 (0.86-1.09) | 0.607 |
| Largest LN diameter | 0.87 (0.77-0.99) | **0.030** |
| LN number (2+3 vs. 1) | 2.04 (0.78-5.31) | 0.145 |
| Delivered dose (Gy) | 1.02 (0.91-1.15) | 0.685 |
| Gleason Score (8+9 vs. 6+7) | 0.53 (0.20-1.43) | 0.213 |
| pT | 1.02 (0.40-2.60) | 0.968 |
| pN | 1.99 (0.70-5.65) | 0.194 |
| SM | 1.53 (0.60-3.91) | 0.373 |
| ECE | 0.77 (0.30-1.96) | 0.581 |
| SVI | 1.05 (0.37-2.95) | 0.932 |
|  |  |  |

Abbreviations: SBRT: stereotactic body radiation therapy; PSA: prostate specific antigen; LN: lymph node; SM: surgical margins; ECE: extra-capsular extension. SVI: seminal vesicles involvement
