# Supplementary figures and images for: Predictive factors for response to salvage stereotactic body radiotherapy in oligorecurrent prostate cancer limited to lymph nodes: a single institution experience
Source: BMC Urol. 2019 Sep 9;19:84. doi: 10.1186/s12894-019-0515-z (PMC6734440; doi:10.1186/s12894-019-0515-z)

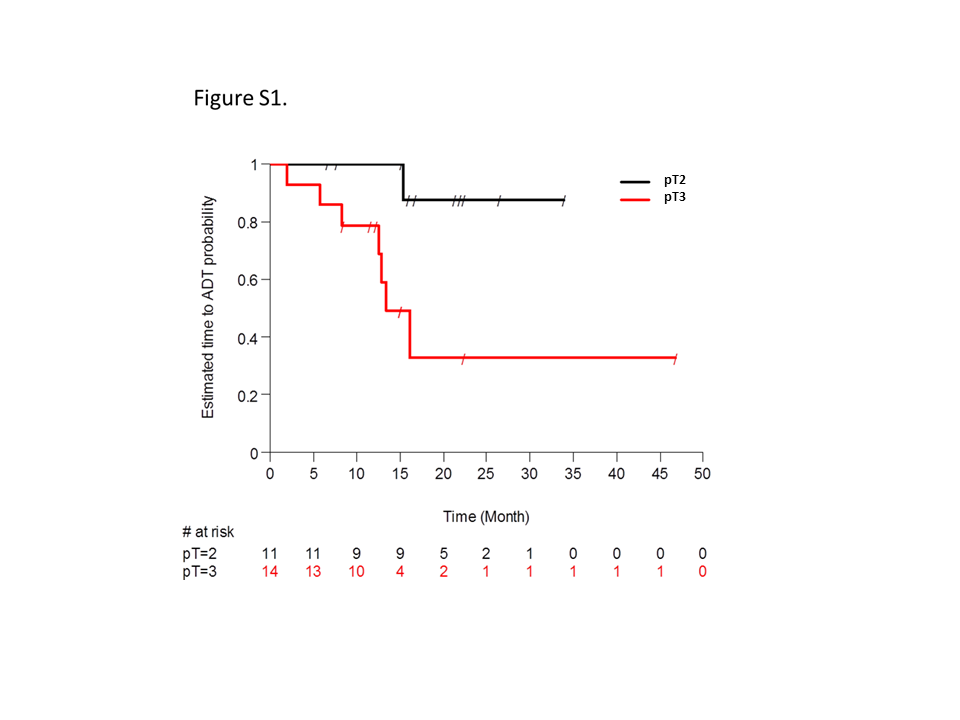

Supplement: Supplementary file 2 — Figure S1. Time to ADT after SBRT according to initial T stage (pT2 vs. pT3). Figure S2. Time to ADT after SBRT according to initial presence/absence of ECE. (ZIP 106 kb) [file 12894_2019_515_MOESM2_ESM.zip › Supplementary Material Figure S1R3.TIF]

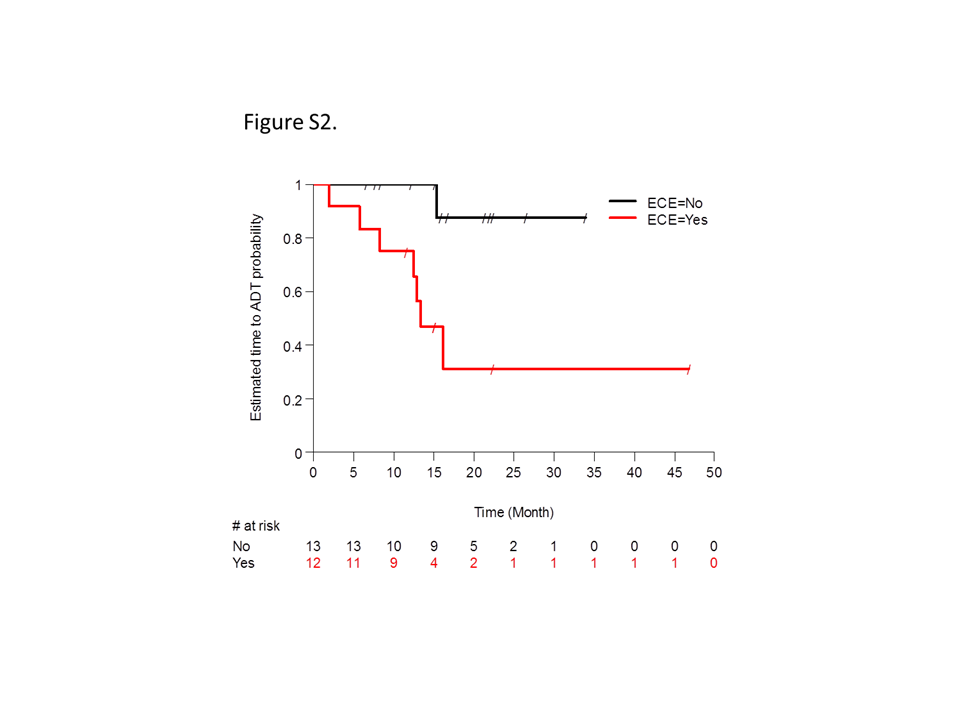

Supplement: Supplementary file 2 — Figure S1. Time to ADT after SBRT according to initial T stage (pT2 vs. pT3). Figure S2. Time to ADT after SBRT according to initial presence/absence of ECE. (ZIP 106 kb) [file 12894_2019_515_MOESM2_ESM.zip › Supplementary Material Figure S2R3.TIF]
